# The mediating effect of resilience on mental health literacy and positive coping style among Chinese empty nesters: A cross-sectional study

**DOI:** 10.3389/fpsyg.2023.1093446

**Published:** 2023-01-25

**Authors:** Li Song, Yujie Wang, Qinghua Zhang, Jinyu Yin, Wei Gan, Siyi Shang, Lingxia Qi, Shengguang Chen, Tongtong Liu

**Affiliations:** School of Nursing, College of Medicine, Huzhou University, Huzhou, China

**Keywords:** mental health literacy, resilience, positive coping style, empty nester, mental health

## Abstract

**Objectives:**

Empty nesters in China have limited mental health literacy (MHL), which may lead to poorer health outcomes. Studies demonstrate that MHL is associated with both resilience and positive coping style. However, the potential mechanism of MHL, resilience and positive coping style remain unclear. Therefore, the study aims to investigate the possible mediating role of resilience in the relationship between MHL and positive coping style.

**Method:**

In this cross-sectional study, a total of 363 empty nesters from Huzhou, China were surveyed in 2022. The Chinese version of Mental Health Literacy Scale (C-MHLS), the Chinese version of 10-item Connor-Davidson Resilience Scale (CD-RISC-10) and the Simplified Coping Style Questionnaire (SCSQ-19) were used to assess MHL, resilience, and positive coping style, respectively.

**Results:**

Positive coping style was significantly correlated with MHL and resilience, and MHL was positively correlated with resilience (*p* < 0.01). MHL can significantly and positively predict the positive coping style, and resilience played a partial intermediary role between MHL and positive coping style, with the intermediary effect of 77.36%.

**Conclusion:**

This study indicates that MHL not only directly affected positive coping style, but also indirectly influences positive coping style by increasing the resilience of empty nesters. The results provide an empirical evidence for the development of intervention programs to improve positive coping style directly and indirectly. Consequently, community health servicers should take targeted measures which focus on MHL and resilience as breakthrough points to stimulate positive coping style of empty nesters, and ultimately achieve their overall well-being.

## 1. Introduction

The World Health Organization predicts that by 2030, one in six people in the world will be aged 60 years or over (World Health Organization, 2021). According to the annual data of the National Bureau of Statistics of China, the number of people who aged over 65 in China has increased sharply from 123 million in 2011 to 201 million in 2021, nearly doubling (Nation Bureau of Statistics, 2021). The explosive growth of the elderly population has brought severe challenges to the Chinese public health, among which empty nesters deserve more attention.

Empty nesters refers to the elderly over 60; have no children or have children, but are not around; live alone or only with their spouses. It can be further divided into absolute empty-nesters (those who do not live with their children in the same city or have no children) and relative empty-nesters (those who live with their children in the same city but do not live together; Yao et al., 2019). Based on the data of China’s seventh census, there are about 119 million empty nesters, and the proportion of empty nesters’ families has reached 44.82%. Hence forecasts, “empty-nest” of Chinese elderly families has become an inevitable trend (National Bureau of Statistics, 2021).

Long-term lacking of care and emotional support will lead to adverse psychological outcomes of empty nesters, such as loneliness, anxiety and depression, and even suffering from mental illness. A survey manifests that up to 54.5% of empty nesters in China have experienced loneliness (Wang et al., 2017). Depression and anxiety are the most common psychological problems of empty nesters in China, with prevalence rates of 38.6 and 41%, respectively, (Wang et al., 2020; Zhang et al., 2020), nearly twice that of the general elderly (23.6 and 22.1%; Su et al., 2011; Li et al., 2014). A study in Shanxi Province proofs that the prevalence of mental disorders among empty nesters is significantly higher than that of non-empty nesters (26.9% vs. 23.5%; Zhang et al., 2019). It can be seen that the current psychological condition of empty nesters is not optimistic at present, which may be closely related to their poor coping style.

Previous studies have pointed out that coping style acts a pivotal part in regulating individual’s psychological state and maintaining mental balance (Li et al., 2022). Coping style refers to a cognitive adjustment behavior method and strategy adopted by an individual to adapt to the requirements of the internal and external environment in the face of stressful events, which can be divided into positive coping style and negative coping style (Lazarus and Folkman, 1984). Su’s research have verified that coping style can directly affect the mental health of empty nesters, and positive coping style is a positive predictor of mental health, while negative coping style is a negative predictor of mental health (Su et al., 2018).

Mental health is a development process in which risk factors and protection factors compete with each other (World Health Organization, 2014). The characteristics of empty nesters are that they need to face the double pressures of material level and spiritual level, which makes them easy to suffer from mental disorders. Old age, infirmity, lack of child support, difficulty in seeking medical care and loneliness are the stressors they face. Based on the stress-coping model by Lazarus and Folkman, stress is the product of the interaction between human and environment. If the internal and external environment stimulation exceeds one’s own coping ability and coping resources, pressure will be generated. Whether a stressor can produce stress after acting on an individual mainly depends on cognitive evaluation and response (Folkman et al., 1986). In this process, mental health literacy (MHL) and resilience can work as protective factors to build cognitive and behavioral resources to cope with psychological stress (Carvalho and Dias, 2021).

Mental health literacy was defined as knowledge and beliefs that contribute to the recognition, management, and prevention of mental illness. It includes the following 6 dimensions: the ability to recognize mental illness; the knowledge of causes and risk factors of mental illness; the knowledge of self-treatment; the knowledge of professional help available; the knowledge of how to seek information related to mental health; the attitudes that promote recognition or appropriate help seeking behavior (Jorm et al., 1997). Several studies suggest that MHL is strongly associated with coping style (Chen, 2016; Li, 2018; Dong, 2019; Carvalho and Dias, 2021), and improving MHL can help enhance coping skills (Katz et al., 2020). The higher the MHL of individuals, the more inclined they are to adopt positive coping style when encountering psychological stress (Li, 2018).

Resilience refers to an individual’ mental ability to actively adjust and adapt in the face of adversity, trauma and other pressures (Schwarz, 2018). A higher level of resilience was related to more positive coping style (Zhao et al., 2020; Xu et al., 2022), and those with high resilience are more inclined to choose positive coping style to deal with stressful events in life, including better problem solving and less avoidant coping (Wu et al., 2020). In addition, Jorm’s research found that increased MHL implies promise in its ability to increase resilience and possibly master more coping strategies (Jorm, 2012). This view has been supported by several studies (Fraser and Pakenham, 2009; Cavanaugh et al., 2021; Sullivan et al., 2021).

Although a large number of studies have revealed the influence of MHL in maintaining and promoting mental health (Brijnath et al., 2016). However, there are few studies on the correlation between MHL and other variables, which variables mediate in the maintenance and promotion of mental health, and how they influence are influenced, remains to be further explored. Consequently, this study aims to explore the relationship between MHL, resilience and positive coping style of empty nesters, so as to provide the basis for ameliorating their mental health.

## 2. Methods

### 2.1. Study population

A multistage stratified random sampling was adopted in Huzhou City, China from March to July, 2022. In the first stage, we used a table of random digit to randomly select one county from five counties in Huzhou City, and then randomly selected five streets from this county. Next, we randomly selected one community from each street, and a total of five communities were extracted. In the second stage, we used the residential information of community health records to randomly select participants under the permission of community health servicers in the selected communities. (1) Inclusion criteria: age ≥ 60; elderly who lived alone or only with their spouse for more than 6 months each year, and their children were absent or childless; awareness was clear, and communication with investigators was barrier-free; volunteered and signed informed consent. (2) Exclusion criteria: had serious physical diseases, mental disorders or cognitive disorders; unable to take care of themselves; children living nearby could take care of the elderly at any time.

### 2.2. Data collection

Before the formal survey, 30 empty nesters were selected by convenience sampling to test the reliability and validity of the instruments, as well as the appropriateness of the survey method and field. During the formal survey, with the assistance of community health servicers, we used a combination of centralized investigation (community health checkups, health lectures, free clinics, etc.), and individual household surveys to conduct on-site investigations. Each data was obtained through a face-to-face survey of about 30 min. The trained researchers used unified instructions to explain the purpose and significance of the study. Prior to the survey, informed consent was obtained from each participant.

### 2.3. Instruments

The general information questionnaire was used to assess the demographic information, including gender, age, residence, degree of education, living style, marital status, monthly income and other information.

Mental health literacy was measured by the Chinese version of Mental Health Literacy Scale (C-MHLS), which was developed by O'Connor and Casey (2015), and translated into Chinese version in 2019 by Ma (2019). It is the first tool to measure all dimensions in the definition of MHL proposed by Jorm. MHL was measured through 35 items in 6 dimensions, which included the ability to recognize mental illness (8 items), the knowledge of causes and risk factors of mental illness (2 items), the knowledge of self-treatment (2 items), the knowledge of professional help available (3 items), the knowledge of seeking information related to mental health (4 items), the attitudes that promote recognition or appropriate help seeking behavior (16 items). 4-point Likert and 5-point Likert scoring method were used in the scale. Items 10, 12, 15 and 20–28 were scored reversely, with a total score of 35–160. Higher scores indicated better MHL. MHLS is a previously verified tool with good internal consistency (Cronbach’s alpha = 0.873). In this study, The Cronbach’s alpha of this scale was 0.879.

*Resilience* was measured by the Chinese version of the 10-item Connor-Davidson Resilience Scale (CD-RISC-10). The CD-RISC-10 was first simplified by Professor Campbell-shills, who extracted 10 items from the 25-item Connor-Davidson Resilience Scale (CD-RISC; Campbell-Sills and Stein, 2007), and then translated and revised into the Chinese version by Zhang et al. (2018). The scale had 10 items and 2 dimensions: strength (5 items) and tenacity (5 items). The scale was scored by 5-point Likert, with “never,” “rarely,” “sometimes,” “often” and “always” being 0–4 points in turn, with a total score of 40. Higher scores represented better resilience. The scale has been evaluated in Chinese older adults, displaying good reliability and validity (Meng et al., 2019). In this study, The Cronbach’s alpha of this scale was 0.904.

*Positive coping style* was measured by the Simplified Coping Style Questionnaire (SCSQ-19). The SCSQ-19 was compiled by Xie (1998) based on a broad coping style questionnaire, and then revised by Zhu et al. (2016) according to the context of the Chinese elderly. This questionnaire consisted of positive and negative style, with the positive coping style assessed in items 1–12, and the negative coping style assessed in items 13–19. The responses ranged from 3 (often) to 0 (not at all). The SCSQ was used to assess coping style among elderly adults in China, and it demonstrated good internal consistency (Cronbach’s alpha = 0.886). In this study, The Cronbach’s alpha of this questionnaire was 0.801.

### 2.4. Statistical analysis

All statistical analyses were carried out using SPSS 24.0 and AMOS 26.0. The analysis strategy was divided into three steps. Firstly, descriptive statistics was used to process sociodemographic data, of which observable variables were presented as the means ± standard deviations (M ± SD) for continuous variables and frequencies and percentages for categorical variables. Secondly, correlation analysis and multiple linear regression analysis were used to explore the direct relationship among variables. Thirdly, we used the bootstrap-based structural equation modeling analysis in AMOS 26.0 software to test the mediating role of resilience between MHL and positive coping style. For the mediation analysis, the positive coping style was considered a dependent variable, MHL was considered an independent variable, and the resilience was entered as mediating variable.

## 3. Results

### 3.1. Demographic characteristics of the participants

Three hundred and seventy-five individuals were enrolled, and 363 completed the questionnaires, for a recovery rate of 96.8%. Eighty-nine cases were absolute empty-nesters, and 274 were relative empty-nesters. The age of these respondents ranged from 60 to 94 years (*M* = 72.69, SD = 8.56) and more than half of them were female (50.7%), married (79.9%), and lived in city (61.4%). The level of education was predominantly primary school (125 cases, 34.4%). Monthly income was mostly <5,000 RMB (62.3%; Table 1).

**Table 1:** Demographic characteristics of the participants (*n* = 363).

Variable	Group	*N* (%)
Gender	Male	179 (49.3)
	Female	184 (50.7)
Age	60–69	141 (38.9)
	70–79	137 (37.7)
	≥80	85 (23.4)
Residence	City	223 (61.4)
	Countryside	140 (38.6)
Education	Illiterate	86 (23.7)
	Primary school	125 (34.4)
	Junior school	93 (25.6)
	High school	30 (8.3)
	Bachelor degree or above	29 (8.0)
Marital status	Married	290 (79.9)
	Single	3 (0.8)
	Divorced	14 (3.9)
	Widowed	56 (15.4)
Living style	Relative empty nest	274 (75.5)
	Absolute empty nest	89 (24.5)
Monthly income	<1,000 RMB	36 (9.9)
	1,000–3,000 RMB	103 (28.4)
	3,000–5,000 RMB	87 (24.0)
	>5,000 RMB	137 (37.7)

### 3.2. Positive coping style, MHL and resilience of participants

The scores of positive coping style, MHL and resilience were shown in Table 2. Furthermore, we compared the average scores of six dimensions of MHL. On the 4-point scoring dimension, the scores from high to low were: the knowledge of causes and risk factors of mental illness (2.76 ± 0.81), the knowledge of self-treatment (2.70 ± 0.54), the knowledge of professional help available (2.63 ± 0.58), and the ability to recognize mental illness (2.61 ± 0.78). On the 5-point scoring dimension, the score of the knowledge of seeking information related to mental health (3.07 ± 1.20) was higher than the attitudes that promote recognition or appropriate help seeking behavior (2.75 ± 0.79).

**Table 2:** The scores of positive coping style, MHL and resilience.

Variable	Score
Positive coping style	21.68 ± 6.28
MHL	95.98 ± 21.46
Ability to recognize mental illness	20.90 ± 6.20
Knowledge of causes and risk factors of mental illness	5.53 ± 1.63
Knowledge of self-treatment	5.40 ± 1.08
Knowledge of professional help available	7.88 ± 1.75
Knowledge of how to seek information related to mental health	12.28 ± 4.80
Attitudes that promote recognition or appropriate help seeking behavior	44.00 ± 21.59
Resilience	24.72 ± 7.07
Tenacity	12.44 ± 3.63
Strength	12.28 ± 3.73

### 3.3. The direct association among MHL, resilience and positive coping style

Correlation analysis (Table 3) showed that positive coping style was significantly and positively correlated with MHL (*r* = 0.554, *p* < 0.01) and resilience (*r* = 0.711, *p* < 0.01). Moreover, there was a significant positive correlation between MHL and resilience (*r* = 0.586, *p* < 0.01).

**Table 3:** Correlations among MHL, positive coping style and resilience.

Variable	Positive coping style	MHL
MHL	0.554^**^	
Resilience	0.711^**^	0.586^**^

^**^p < 0.01.

Multiple linear regression models were constructed to expose the effects of resilience and MHL on positive coping style (Table 4). Two dimensions of resilience, namely tenacity and strength were significantly associated with positive coping style (*p* < 0.001, *p* = 0.038, respectively). “knowledge of self-treatment” and “attitudes that promote recognition or appropriate help seeking behavior” of MHL were significantly associated with positive coping style (*p* = 0.012, *p* = 0.001, respectively). However, other dimensions of MHL had no statistical significant effect on positive coping style (*p* > 0.05).

**Table 4:** The effects of MHL and resilience on positive coping style.

Dependent variable	Independent variable	*B*	S.E.	*β*	*t*	*p*
Positive coping style	MHL					
	Ability to recognize mental illness	−0.048	0.055	−0.048	−0.881	0.379
	Knowledge of causes and risk factors of mental illness	0.066	0.148	0.017	0.448	0.654
	Knowledge of self-treatment	0.588	0.232	0.101	2.538	0.012^*^
	Knowledge of professional help available	0.214	0.177	0.060	1.210	0.227
	Knowledge of how to seek information related to mental health	0.043	0.070	0.033	0.616	0.538
	Attitudes that promote recognition or appropriate help seeking behavior	0.073	0.021	0.146	3.054	0.001^*^
	Resilience					
	Tenacity	0.809	0.120	0.469	6.740	0.000^*^
	Strength	0.242	0.116	0.144	2.082	0.038^*^

*p < 0.05.

### 3.4. Mediating effect of resilience of empty nesters on MHL and positive coping style

#### 3.4.1. Common method variance

We used Harman’s one-factor method to test the common method deviation. All the items of the scale used to measure MHL, resilience and positive coping style were included in SPSS 24.0 software for exploratory factor analysis. The results showed that 12 factors with characteristic root > 1 were extracted, and the maximum factor variance explanation rate was 15.15% (< 40%), so there was no serious common method deviation in this study (Podsakoff et al., 2012).

#### 3.4.2. Mediary model estimation

In this study, the analysis was performed by a bootstrap-based structural equation model to test the mediation effect of the resilience on the correlation between MHL and positive coping style. Several indexes were calculated to evaluate the model fit to the data: chi-square statistic (*χ^2^*), *χ^2^*/df, root mean square error of approximation (RMSEA), adjusted goodness-of-fit index (AGFI), comparative fitting index (CFI), goodness-of-fit index (GFI), and normed fit index (NFI).

**Figure 1:**
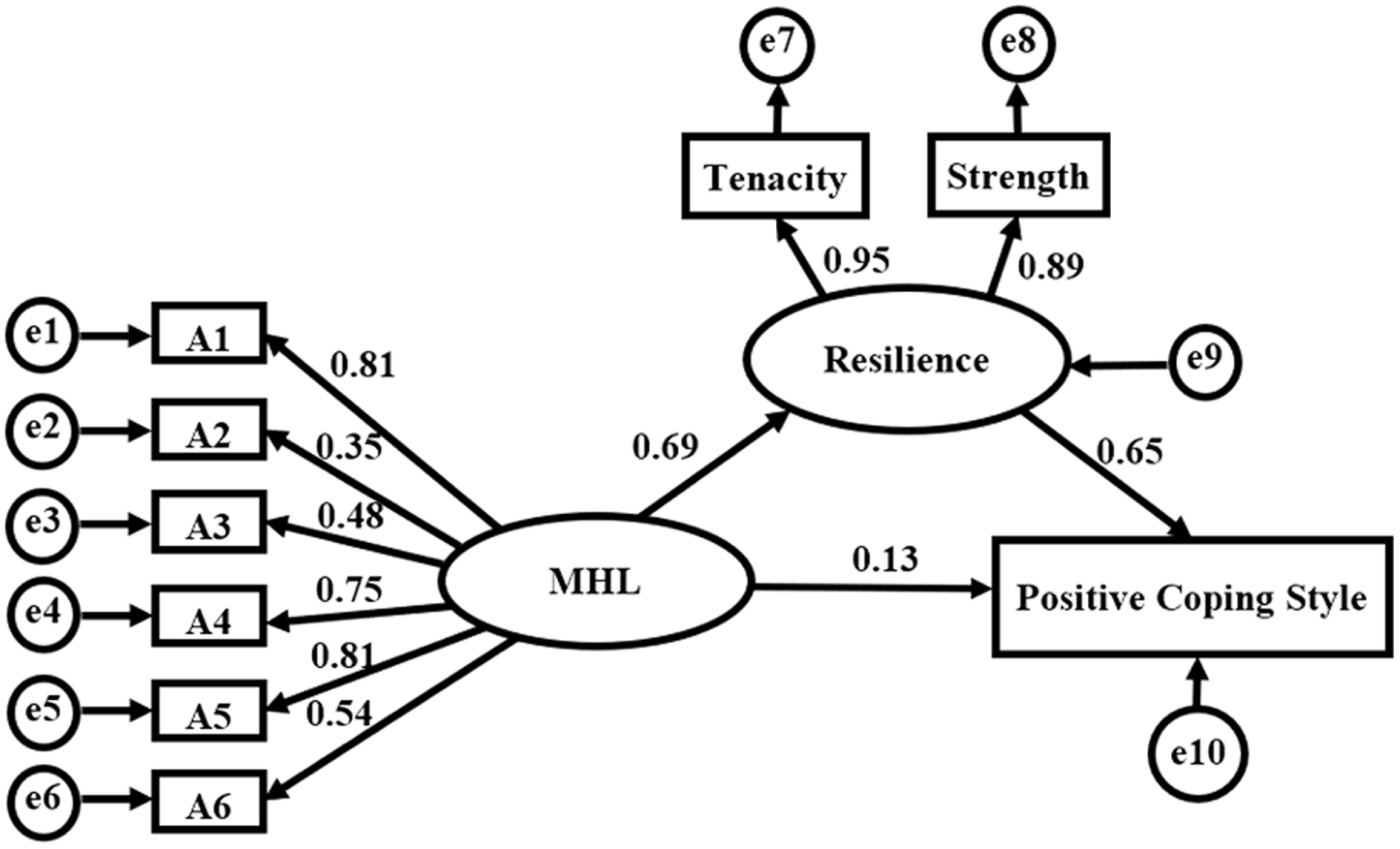
The structural equation model of MHL and resilience affecting positive coping style among empty nesters. A1: the ability to recognize mental illness; A2: the knowledge of causes and risk factors of mental illness; A3: the knowledge of self-treatment; A4: the knowledge of professional help available; A5: the knowledge of how to seek information related to mental health; A6: the attitudes that promote recognition or appropriate help seeking behavior.

The fitting results of the model was suggestive of all fitting indexes of the model met the standard, indicating that the fitting effect of the model was good. See Table 5 for the results. See Figure 1 for the model.

**Table 5:** Model fitting index.

Indice	*χ*^2^/*df*	AGFI	CFI	GFI	NFI	RMSEA
Reference range	1–3	>0.90	>0.90	>0.90	>0.90	<0.08
Fitting result	2.605	0.932	0.974	0.962	0.959	0.067

#### 3.4.3. Mediation effect test

According to the structural equation model, the preliminary judgment was that a positive coping style mediation path existed, but the mediation effect (path coefficient of the product) needed further verification. Bootstrapping was performed to confirm the mediation effect, taking 95% confidence interval (CI) and sampling number of 5,000. The results were shown in Table 6. The 95%CI of the direct and indirect effects of MHL on positive coping style did not contain 0, indicating that the mediation effect model was established. As seen in Figure 1 and Table 6 that the indirect effect of MHL on positive coping style through resilience is 0.451, and the total effect value is 0.584, with the mediating effect accounting for 77.36% of the total effect.

**Table 6:** The mediating effect of resilience on MHL and positive coping style.

Variable	Coefficient	SE	*p*	95%CI
MHL → resilience → positive coping style				
Total effect	0.584	0.039	<0.001	0.507–0.657
Direct effect	0.133	0.060	0.037	0.008–0.247
MHL → resilience	0.689	0.037	<0.001	0.613–0.759
Resilience → positive coping style	0.655	0.049	<0.001	0.559–0.751
Indirect effect	0.451	0.045	<0.001	0.371–0.552

## 4. Discussion

As far as we know, this is the first study to investigate the MHL among empty nesters, and analysis the mediating role of resilience on MHL and the positive coping style using a structural equation model. Findings highlighted that positive coping style is influenced by both MHL and resilience, and the indirect pathway is more influential. The scores of MHL, resilience and positive coping style of empty nesters in this study belong to the lower middle level, which were consistent with previous studies (Kim et al., 2017; Cao et al., 2018; Hao et al., 2021).

Empty nesters with enough MHL are more likely to adopt positive coping styles. On the one hand, characters with high MHL possess sufficient mental health knowledge, so that they can identify mental diseases accurately in the early stage (Yu et al., 2016). Furthmore, it is easier for them to seek effective information related to mental health, and master certain mental health first aid ability, so they are more capable of taking positive coping styles (E.g., using mental health services.) to manage their own and others’ mental health (Mackenzie and Pankratz, 2022). On the other hand, MHL is the strongest factor affecting mental health attitude (Lee et al., 2020). Characters with insufficient MHL usually exist a higher degree of stigma towards mental illness (Svensson and Hansson, 2016). They are more inclined to adopt negative coping styles, such as concealing their illness and avoiding medical treatment (Conner et al., 2010), which not only hinders themselves from seeking professional help, but also make them more likely to treat psychiatric patients with discrimination instead of assistance (Schnyder et al., 2017).

In our study, MHL is proved to influence coping style through resilience, which is consistent with the findings of Sun et al. (2021). When facing with psychological pressure, people with high MHL can quickly adjust their mentality and strengthen their beliefs, so that they can recover from troubles as soon as possible. During the period, their adaptability is gradually improved, which provides a favorable prerequisite for the development of resilience (Fraser and Pakenham, 2009). Previous studies have revealed that resilience is significantly associated with positive coping style (Luo, 2013; Yang, 2015). Someone with high resilience tend to keep a positive and optimistic attitude towards life, and usually regard stress as a controllable event (Wu et al., 2021). Meanwhile, they can understand the cruciality of positive coping style more clearly, and their self-confidence and problem-solving ability are relatively strong (Steinhardt and Dolbier, 2008). All these are beneficial to encourage them to adopt positive coping styles under psychological pressure, effectively overcome the adverse effects of negative emotions, thus reducing or even eliminating symptoms.

In China, poor MHL among empty nesters is alarming. Studies substantiates that MHL will influence empty nesters’ ability to comprehensively apply mental health knowledge, skills and attitudes to deal with mental diseases (Wei et al., 2015), which in turn affects their coping style and mental health outcomes. However, it is remarkable that interventions targeting people with low MHL can be effective in improving their mental health, such as mental health education activities, anti-stigma campaigns and mental health first aid trainings (Jorm, 2012). In addition, as a positive personality trait that can well regulate emotions and psychological resources, resilience may lead to better MHL, thereby enhancing their positive coping style (Cao et al., 2020). Therefore, community health servicers should focus on the MHL and resilience of empty nesters, then take these two intervenable and changeable factors as entry points to develop intervention programs that directly or indirectly improve their positive coping style. Since positive coping style is an important positive predictor of empty nesters’ mental health, promoting positive coping style will help achieve their overall well-being. It will also imperceptibly enhance empty nesters’ awareness of their own mental health care, mobilize their initiative, and encourage them to be the first responsible person for their own mental health, thus providing a feasible path to cope with the current situation of aging and declining birthrate in China.

## 5. Limitations

Several limitations of this research should be noted. First, the sample population of empty nesters included only a few randomly selected communities in a city, which may introduce bias and limit the universality of the research results. Accordingly, it is indispensable to conduct future research with empty nesters from different regions. Second, it is difficult to draw a conclusion on the causal relationship between the research variables because of the cross-sectional design used in this study. Third, the data were collected through self-report questionnaires, which may be subject to reporting bias.

## 6. Conclusion

This study bears out that resilience is an important mediator in the relationship between MHL and positive coping style of empty nesters. The results provide an empirical evidence for the development of intervention programs to improve positive coping style directly and indirectly. Consequently, community health servicers should take targeted measures which focus on MHL and resilience as breakthrough points to stimulate positive coping style of empty nesters, and ultimately achieve their overall well-being.

## Data availability statement

The original contributions presented in the study are included in the article/supplementary material, further inquiries can be directed to the corresponding author.

## Ethics statement

The studies involving human participants were reviewed and approved by the ethics committee of the School of Nursing, College of Medicine, Huzhou University (2022-03-14). The patients/participants provided their written informed consent to participate in this study.

## Author contributions

QZ was involved in research design and critical revision for intellectual content of the manuscript. LS completed the first draft of the manuscript, submitted the manuscript for publication. YW, JY, and WG contributed to assist in the development of the study protocol. SS and LQ assisted with participant enrollment and consent, and worked out the data acquisition plan. All authors commented on previous versions of the manuscript, read and approved the final manuscript.

## Funding

This work was supported by the National Social Science Fund of China (22BGL251), China Scholarship Council (202008330254), Scientific Research Fund of Zhejiang Provincial Education Department (Y202250172) and Postgraduate Research and Innovation Project of Huzhou University (2022KYCX68).

## Conflict of interest

The authors declare that the research was conducted in the absence of any commercial or financial relationships that could be construed as a potential conflict of interest.

## Publisher’s note

All claims expressed in this article are solely those of the authors and do not necessarily represent those of their affiliated organizations, or those of the publisher, the editors and the reviewers. Any product that may be evaluated in this article, or claim that may be made by its manufacturer, is not guaranteed or endorsed by the publisher.
